# Plasmonic bandgap in random media

**DOI:** 10.1186/1556-276X-8-324

**Published:** 2013-07-16

**Authors:** Valentina V Zhurikhina, Michael I Petrov, Oksana V Shustova, Yuri P Svirko, Andrey A Lipovskii

**Affiliations:** 1 Department of Nanostructures Physics and Technology, St. Petersburg State Polytechnical University, Polytechnicheskaya 29, St. Petersburg 195251, Russia; 2 Department of Condensed Matter Physics, St. Petersburg Academic University, Khlopina 8/3, St. Petersburg 195220, Russia; 3 Institute of Photonics, University of Eastern Finland, Yliopistokatu 7, P.O. Box 111, Joensuu, 80101, Finland

**Keywords:** Metal-dielectric nanocomposite, Surface plasmon polariton, Drude metal, Silver, Dispersion, 78.67.Sc, 81.05.Ni, 71.36.+c

## Abstract

We present a dispersion theory of the surface plasmon polaritons (SPP) in random metal-dielectric nanocomposite (MDN) consisting of bulk metal embedded with dielectric inclusions. We demonstrate that embedding of dielectric nanoparticles in metal results in the formation of the plasmonic bandgap due to strong coupling of the SPP at the metal-vacuum interface and surface plasmons localized at the surface of nanoinclusions. Our results show that MDN can replace metals in various plasmonic devices, which properties can be tuned in a wide spectral range. Being compatible with waveguides and other photonic structures, MDN offers high flexibility in the plasmonic system design.

## Background

The resonant coupling of light to oscillations of the free electron density near the metal surface, surface plasmons (SP), gave birth to a variety of advanced applications ranging from sensing to nonlinear optics. SPs are bound to the metallic surface, i.e., at the frequency of the surface plasmon resonance, light field exponentially decays in neighboring media. Since the decay length of SPs is two orders of magnitude smaller than the wavelength of the light in air, they can be employed for subwavelength localization of light. The guiding of light in plasmonic structures is possible via surface plasmon polaritons (SPP) that can propagate in periodical arrays of metal nanoparticles embedded in dielectrics. The multiple scattering of the SPPs off the periodic corrugation leads to the Bragg-like plasmon modes [1,2] and to the plasmonic band gaps [1,3], i.e., they do not allow the SPP in a certain interval of wavelengths. When metal nanoparticles are placed into dielectric in a random fashion, e.g., in metal island films [4,5], nanoporous metal films [6], and metal-dielectric nanocomposite (MDN) [7-10], no SPP bandgaps have been observed. The optical properties of these materials dominated by SPs localized on individual metal nanoparticles are well studied [11,12]; however, much less attention was paid to the behavior of SPP propagating at the MDN-dielectric interface. In this paper, we present the theory of SPP in MDN based on noble metals with random distribution of the dielectric inclusions. We demonstrate that when the metal volume content is high, the coupling of propagating and localized at metal-inclusion interface plasmon modes results in the formation of the SPP bandgap in such random media. By using Drude model for dielectric function of the metal, we develop dispersion theory of the SPP at the MDN-vacuum surface. We demonstrate that in silver, bandgap persists when dielectric properties of the metal are described by experimental data. The presence of the SPP bandgap indicates that the MDN can replace metals in various plasmonic structures that will benefit from the tunability of the MDN properties.

## Methods

We consider the interface between a dielectric with a real positive dielectric constant *ϵ*_1_ (*z* < 0) and a MDN with a frequency-dependent complex dielectric function *ϵ*_2_(*ω*) *n* (*z* > 0). The electric filed associated with SPP propagating along *x*-axis can be presented in the following form:

(1) $$\begin{array}{l} E=E^{\left(1\right)}\exp\left(ik_{\mathrm{SPP}}x-\delta_{1}z\right),\;z>0\\ E=E^{\left(2\right)}\exp\left(ik_{\mathrm{SPP}}x+\delta_{2}z\right),\;z<0 \end{array}$$

where [13]

(2) $$k_{\mathrm{SPP}}^{2}=\frac{\omega^{2}}{c^{2}}\cdot \frac{\epsilon_{1}\epsilon_{2}}{\epsilon_{1}+\epsilon_{2}},\;\delta_{1,2}^{2}=-\frac{\omega^{2}}{c^{2}}\frac{\epsilon_{1,2}^{2}}{\epsilon_{1}+\epsilon_{2}}.$$

One can observe from Equations 1 and 2 that SPP is allowed at Re(*ϵ*_2_(*ω*) + *ϵ*_1_) < 0 when Re(*k*_*SPP*_) ≠ 0 and Im(*δ*_1,2_) = 0. The condition Re(*ϵ*_2_(*ω*) + *ϵ*_1_) = 0 corresponds to the excitation of the surface plasmon [1,13]. If Re(*ϵ*_2_(*ω*)) > 0, SPP is forbidden; however, a transversal bulk plasmon polariton (BPP) with wave vector $k_{\mathrm{BPP}}=\left(\omega /c\right)\sqrt{\epsilon_{2}\left(\omega \right)}$ can propagate at *z* > 0. If 0 > Re(*ϵ*_2_(*ω*)) > − *ϵ*_1_, no propagating electromagnetic perturbations are allowed, i.e., the energy of the incident light wave is transferred to the localized plasmons.

When the concentration of dielectric inclusions *g* is relatively low (*g* < 0.15), the dielectric constant of the MDN can be described in the framework of Maxwell Garnett approach [14] for dielectric inclusions in metal that yields

(3) $$\epsilon_{\mathrm{eff}}\left(\omega \right)=\epsilon \left(\omega \right)\frac{\left(2\epsilon \left(\omega \right)+\epsilon_{d}\right)+2g\left(\epsilon_{d}-\epsilon \left(\omega \right)\right)}{\left(2\epsilon \left(\omega \right)+\epsilon_{d}\right)-g\left(\epsilon_{d}-\epsilon \left(\omega \right)\right)}.$$

Assuming that the permittivity of metal can be described in terms of the Drude model with no scattering,

(4) $$\epsilon \left(\omega \right)=1-\frac{\omega_{p}{}^{2}}{\omega^{2}},$$

where *ω*_p_ is the plasma frequency, the effective dielectric function can be presented as

$$\epsilon_{\mathrm{eff}}\left(\omega \right)=\frac{2\left(1-g\right)}{2+g}\frac{\Omega_{\mathrm{TO}}^{2}}{\Omega_{\mathrm{LO}}^{2}}\frac{\left(\omega^{2}-\omega_{p}^{2}\right)\left(\omega^{2}-\Omega_{\mathrm{LO}}^{2}\right)}{\omega^{2}\left(\omega^{2}-\Omega_{\mathrm{TO}}^{2}\right)}$$

(5) $$\Omega_{\mathrm{TO}}^{2}=\frac{2\omega_{p}^{2}\left(1-g\right)}{2+\epsilon_{d}-g\left(\epsilon_{d}-1\right)},\;\Omega_{\mathrm{LO}}^{2}=\frac{\omega_{p}^{2}\left(2+g\right)}{2+\epsilon_{d}+2g\left(\epsilon_{d}-1\right)}.$$

One can see from Equation 5 that the effective dielectric function has singularities at *ω =* 0 and *ω = Ω*_TO_. The singularity at *ω =* 0 is a conventional ‘metal’ one, while the singularity at *ω = Ω*_TO_ corresponds to the collective oscillations of the conduction electrons at the surface of dielectric nanoparticles incorporated into the metal matrix, i.e., localized surface plasmon resonance at the metal-dielectric interface. Frequency *Ω*_LO_ corresponds to the excitation of the longitudinal phonons in the GMN.

**Figure 1:**
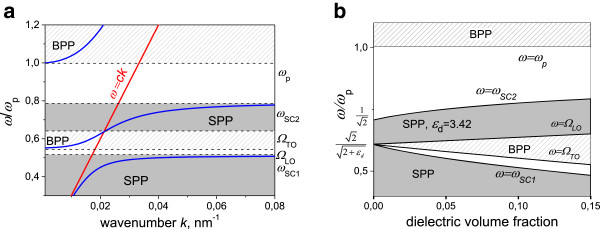
**Dispersion relation for plasmon polaritons and map of electromagnetic modes for Drude MDN without scattering. (a)** Dispersion relation for plasmon polaritons at *ω*_p_ = 10^16^ s^−1^, *g* = 0.1 and *ϵ*_d_ = 3.42 (blue line). The light line *ω* = *ck* is also shown. **(b)** Map of the electromagnetic modes in the *g-ω* plane. SPP and BPP exist in gray and hatched areas, respectively.

The surface plasmon frequency *ω*_SC_ at the MDN-vacuum interface can be found from the condition *ϵ*_eff_(*ω*_SC_) = −1. Solution of this equation yields

(6) $$\begin{array}{l} \omega_{\mathrm{SC}1,2}^{2}=4\left(1-g\right)\omega_{p}^{2}[6+\epsilon_{d}+g\left(2\epsilon_{d}-3\right)\\ \;{\pm \sqrt{{\left[2+\epsilon_{d}+g\left(2\epsilon_{d}+1\right)\right]}^{2}-8\epsilon_{d}{\left(1-g\right)}^{2}}]}^{-1}, \end{array}$$

i.e., two surface plasmon frequencies can exist. In pure metal (*g* = 0), SPP can propagate along the metal/vacuum interface at $\omega <\omega_{p}/\sqrt{2}$[13]. However, at a finite dielectric content, *g >* 0, the SPP band splits into two, i.e., SPP is allowed at *ω*_LO_*< ω < ω*_SC2_ and *ω < ω*_SC1_. These two SPP bands are separated by a BPP band from *Ω*_LO_ to *Ω*_TO_ and a forbidden gap between *ω*_SC1_ and *Ω*_TO_ (see Figure 1)*.* Thus, strong coupling between SPP at the metal vacuum interface and localized surface plasmons at the surface of randomly distributed dielectric nanoinclusions results in the formation of the plasmonic bandgap, which is conventionally observed in plasmonic crystals.

## Results and discussion

The dispersion relation for propagating electromagnetic modes in Drude MDN with dielectric volume fraction *g* = 0.1 and *ϵ*_d_ = 3.42 is shown in Figure 1a. Figure 1b shows the map of collective excitations in Drude MDN in the ‘*ω*-*g*’ plane at *ϵ*_d_*=* 3.42. One can observe two SPP bands, the BPP band, and the forbidden gap separated by frequencies *Ω*_LO_, *Ω*_TO_, and *ω*_SC1_*.* The upper limit of the higher SPP zone is *ω*_SC2_. There also exists the second BPP frequency range for *ω* >*ω*_p_. The width of both SPP and BPP bands increases with the increase of dielectric contained in MDN. The latter was earlier demonstrated by N. Stefanou and coauthors [15] for mesoporous metals. Our calculations also showed that the higher the permittivity of dielectric inclusions in MDN, the broader the upper SPP band and the bigger the downshift of the SPP forbidden gap.

When *g* → 0, the upper MDN surface plasmon frequency $\omega_{\mathrm{SC}2}\to \omega_{p}/\sqrt{2}$, that is, the surface plasmon frequency at metal-air interface, while *Ω*_LO_, *Ω*_TO_, and *ω*_SC1_ approach $\omega_{p}\sqrt{2/\left(2+\epsilon_{d}\right)}$, that is, the SP resonance of a single dielectric cavity in metal matrix [15]. At *ϵ*_d_ > 2, the frequencies *Ω*_LO_, *Ω*_TO_, and *ω*_SC1_ are lower than *ω*_SC2_, and BPP zone and the conventional metal SPP band at *ω < ω*_SC2_ splits by two (see Figure 1b). At *ϵ*_d_ < 2, the *Ω*_LO_, *Ω*_TO_, and *ω*_SC1_ are higher than *ω*_SC2_, and the conventional metal SPP band at *ω < ω*_SC2_ remains intact, however, the second SPP band appears at *ω*_LO_*< ω < ω*_SC2_. At $\epsilon_{d}=2\text{and}g=0,\Omega_{\mathrm{LO}}=\Omega_{\mathrm{TO}}=\omega_{\mathrm{SC}1}=\omega_{\mathrm{SC}2}=\omega_{p}/\sqrt{2}$.

It is worth noting that the dielectric dispersion should change the characteristic frequencies that will lead to the frequency shift of all bands and, in the case of strong dispersion, could possibly result in broadening or vanishing of the second SPP band. But for the most optically transparent dielectrics, their dispersion is negligible compared to the metal one. In this paper we neglect the dielectric dispersion that is valid, for example, for glasses in the visible and near-infrared range.

**Figure 2:**
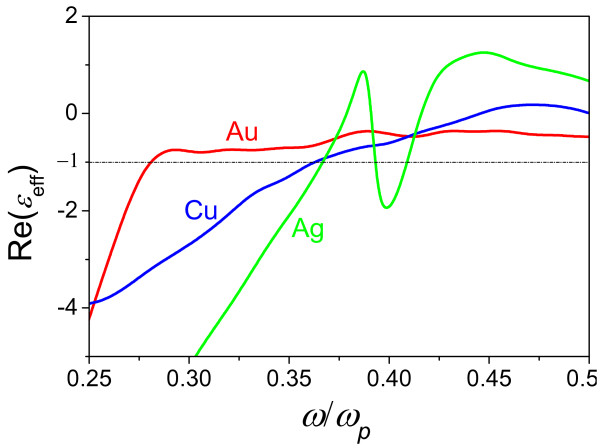
**Real part of the effective dielectric function for the Au-, Cu- and Ag-based MDNs.** The real part of the effective dielectric function *ϵ*_eff_(*ω*) for the Au-, Cu- and Ag-based MDNs is calculated using Johnson and Christy [16] data and Equation 3 at *ϵ*_d_ = 3.42 at *g* = 0.1.

Although Drude approximation satisfactorily describes the optical properties of noble metals, the dissipation of light energy may essentially influence the electromagnetic modes in MDN. When the imaginary part of the metal permittivity is nonzero, the effective permittivity of the MDN is also complex, $\epsilon_{\mathrm{eff}}\left(\omega \right)=\epsilon_{\mathrm{eff}}^{I}\left(\omega \right)+i\epsilon_{\mathrm{eff}}^{\mathrm{II}}\left(\omega \right)$; however, the SPP on the vacuum-MDN interface is allowed (i.e., $\epsilon_{\mathrm{eff}}^{I}\left(\omega \right)<-1$) even at relatively high dielectric volume fractions. In particular, it has been shown both experimentally and theoretically that the gold-based MDN with dielectric volume fraction of *g* ≈ 0.5 supports SPP [6,10]. Figure 2 presents the dependence of the real part of the effective dielectric function of MDN based on noble metals. By using the data for the complex dielectric function from Johnson and Christy [16], one can obtain that at *ϵ*_d_ = 3.42 (flint glass) and *g* = 0.1, the SPP is allowed in Au-, Cu- and Ag-based MDNs; however, the second SPP band occurs in the Ag-based MDN only. However, it is worth noting that even in the silver-based MDN, the SPP band splitting vanishes at *ϵ*_d_ < 2.25.

**Figure 3:**
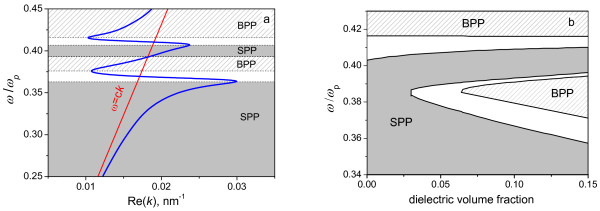
**Dispersion curve for silver-based MDN and map of electromagnetic modes. (a)** The dispersion curve for silver-based MDN at *ω*_p_ = 1.39·10^16^ s^−1^, *g* = 0.1 and *ϵ*_d_ = 3.42 (blue line). The light line *ω*=*ck* is also shown. **(b)** Map of the electromagnetic modes in the *g-ω* plane. SPP and BPP exist in gray and hatched areas, respectively.

Figure 3a shows the plasmon polariton dispersion in silver-based MDN at *g* = 0.1 and *ϵ*_d_ = 3.42 calculated using measured metal permittivity and plasma frequency [16]*ω*_p_ = 1.39·10^16^s^−1^. One can observe from Figure 3a that at Re(*k*) *> ω*/*c*, there exist two SPP and two BPP bands.

Figure 3b shows the map of collective excitations in silver-based MDN on the *ω-g* plane at *ϵ*_d_ = 3.42. One can observe that the shape and size of the gray area in which SPP is allowed is similar to that for Drude MDN (see Figure 1); however, the nonzero imaginary part of the dielectric permittivity of silver results in vanishing of the SPP bandgap at *g* < 0.03. Thus, only one surface plasmon polariton band exists at *g* < 0.03.

## Conclusions

We demonstrate that SPP bandgap can exist not only in plasmonic crystals but also in MDN with low dielectric volume fraction, i.e., when dielectric nanoinclusions are distributed in a random fashion in metal host. In the MDN, the SPP bandgap arises due to strong coupling between SPP at the metal-dielectric interface and plasmons localized on dielectric nanoinclusions allowing one to tailor the plasmonic properties by changing the dielectric content. By using Maxwell-Garnett model, we calculated effective dielectric permittivity of the MDN using both Drude model and Johnson and Christy data for complex dielectric function of metal. We showed that dissipation caused by the scattering of conduction electrons in metal may result in vanishing plasmonic bandgap in noble metal-based MDN. However, at refractive index of dielectric inclusions *n >* 1.5, the plasmonic bandgap survives in Ag-based MDN offering high flexibility in the plasmonic system design.

## Abbreviations

MDN: Metal-dielectric nanocomposite; SP: Surface plasmons; SPP: Surface plasmon polaritons.

## Competing interests

The authors declare that they have no competing interests.

## Authors’ contributions

VZh developed the models used and performed the data analysis. MP performed the dispersion related analysis. OSh obtained the numerical results and found the fitting parameters. YuS performed the analytical analysis. AL supervised the whole work starting from analysis to the interpretation of results. All authors read and approved the final manuscript.

## Authors’ information

VZh holds associate professor position at St. Petersburg State Polytechnical University. MP holds PhD degree at St. Petersburg Academic University. OSh is a PhD student at St. Petersburg State Polytechnical University. YuS holds DrSci degree and professor position at the University of Eastern Finland. AL holds DrSci degree and professor positions at St. Petersburg Academic University and St. Petersburg State Polytechnical University.
